# Tenosynovial Giant Cell Tumor Mimicking Acute Septic Arthritis of the Hip: A Case Report

**DOI:** 10.5435/JAAOSGlobal-D-22-00011

**Published:** 2022-06-06

**Authors:** Evan Honig, Andrew Harris, Samir Sabharwal, Adam Levin, Erin Honcharuk

**Affiliations:** From the Weill Cornell Medical College, New York, NY (Honig) and the Department of Orthopaedic Surgery, The Johns Hopkins University, Baltimore, MD (Dr. Harris, Dr. Sabharwal, Dr. Levin, and Dr. Honcharuk).

## Abstract

A 12-year-old boy presented to the pediatric emergency department with a 5-day history of atraumatic, progressively worsening right hip pain and inability to ambulate. He was afebrile and had elevated inflammatory markers (Erythrocyte Sedimentation Rate [ESR]: 42 mm/hr, C-Reactive Protein [CRP]: 6.6 mg/dL) with a normal white blood cell count of 6050 cells/mm^3^. Given the clinical concern for septic arthritis, joint aspiration of the right hip was done and demonstrated a bloody appearance with a WBC count of 54,999 cells/mm^3^ and RBC count of 7,000 cells/mm^3^. MRI of the right hip demonstrated an intra-articular mass suggestive of tenosynovial giant cell tumor/pigmented villonodular synovitis. Subsequent biopsy and excision of the mass confirmed the diagnosis.

The acute presentation of tenosynovial giant cell tumor with features mimicking septic arthritis is uncommon. This rare presentation of an already uncommon diagnosis should be considered in a child with an equivocal presentation for severe hip pain because misdiagnosis may lead to unnecessary or inadequately planned surgical treatment of the condition.

Tenosynovial giant cell tumor (TGCT)/pigmented villonodular synovitis is a locally aggressive neoplastic disease that affects the synovial joints and tendon sheaths.^1,2^ The reported incidence is approximately 1.8 cases per million, with most cases occurring in patients between the ages of 20 and 50 years.^1,3,4^ The incidence in the pediatric population is even more rare, with only a limited number of cases reported in the literature. Of the reported cases in children, most involve the knee joint, although there have been cases involving various other joints such as the hip, ankle, and sacroiliac joints or widespread multiple joint involvement.^5,6 7 8 9 10 11 12 13 14^

Typically, TGCT of a large joint presents as chronic joint pain and swelling. A case series of 17 pediatric patients with TGCT demonstrated that most of the patients had chronic, insidious joint pain and symptoms before diagnosis and treatment of TGCT.^14^ However, in the few cases of TGCT of the hip reported in the pediatric population, patients have reported acute, rapidly progressive hip pain. In two separate case reports, an 8-year-old and a 9-year-old patient both presented with acute hip pain, suspicious for possible septic arthritis. After equivocal infectious workups, these patients underwent open excision of their intra-articular hip masses, with an eventual diagnosis and treatment of TGCT in both patients.^8,9^

Here, we present the case of a 12-year-old boy with TGCT of the right hip, who presented with acute worsening hip pain.

The patient and his family were contacted for their permission to publish details of his case in a peer-reviewed scientific journal, and they consented.

## Case Report

A 12-year-old boy presented to the pediatric emergency department with a 5-day history of atraumatic, progressively worsening right hip pain. The patient and his parents recall a possible fall from a swing set around the time the pain started, but they were not able to definitively correlate this low-energy traumatic event with his symptoms. He was initially seen at an outside emergency department, where his erythrocyte sedimentation rate (ESR), C-reactive protein (CRP), white blood cell count (WBC), and radiographs of the hip were normal. He was subsequently diagnosed with transient synovitis. The night following this initial emergency department visit, he was awakened from sleep by his right hip pain and was unable to bear weight on the right lower extremity. He returned to the outside emergency department again the next day reporting of worsened right hip pain and was discharged with additional nonsteroidal anti-inflammatories. He was then seen by an outside orthopaedic surgeon, who recommended that the patient present to the pediatric emergency department (level 1 pediatric trauma center) for additional evaluation given the clinical concern for septic arthritis.

At the time of presentation to our institution's emergency department, the patient reported notable right hip pain making him unable to bear weight and waking him from sleep. Of note, he had been afebrile since the onset of his pain. On examination, the patient refused to bear weight on the right lower extremity and held the right hip in flexion, abduction, and external rotation. Interestingly, within a moderate arc of hip range of motion, he had minimal pain. However, at the extremes of internal rotation, especially with the hip flexed, he had increased pain. He was also acutely tender over the medial groin region and the adductor tendons. He was afebrile (T: 37°C), and his WBC count was within normal limits (6,050 WBCs/mm^3^). However, ESR and CRP were elevated to 42 mm/hr and 6.6 mg/dL, respectively (institutional reference ranges: ESR 1 to 15 mm/hr; CRP <0.5 mg/dL).

**Figure 1:**
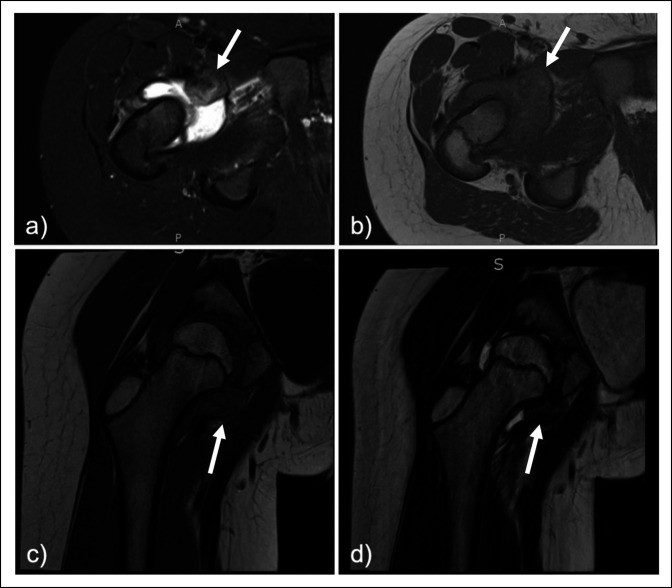
Radiographs showing short-time inversion recovery (STIR) axial (**A**), T1-weighted axial (**B**), T1-weighted coronal (**C**), and T2-weighted coronal (**D**) MR images of the hip demonstrating a 2.5 × 2.1 × 1.4 cm heterogenous mass within anterior-inferior joint space, with hypointensity in both T1 and T2 with enhancement (arrows). Heterogenous moderate right joint effusion with synovitis is also seen.

Radiographs and ultrasonography were done, which both suggested an effusion at the hip joint. An ultrasound-guided hip arthrocentesis was done, and the fluid was grossly bloody with a WBC count of 54,999 cells/mm^3^, a RBC count of 7,000 cells/mm^3^, and a negative Gram stain. Given the equivocal nature of the patient's examination and laboratory test results, MRI with and without contrast of the right hip was ordered for additional evaluation. Imaging demonstrated a heterogeneous intra-articular mass with a heterogenous moderate hip effusion and synovitis suggestive of localized/nodular TGCT (Figure 1). A CT-guided biopsy was done, with pathology demonstrating blood with scant fragments of tenosynovial tissue, which was insufficient for definitive diagnosis. Given the high clinical suspicion for TGCT, he underwent open excisional biopsy with partial synovectomy through an anterior approach. Pathology of the right hip mass demonstrated an entirely necrotic/infarcted TGCT and synovium with chronic synovitis and reactive changes.

At the 2-week follow-up, the patient was recovering well, walking without any assistive devices and without a limp. He had minimal pain and had discontinued analgesics. Two months after surgery, he had no reports and had returned to full activity without limitations and with no pain.

## Discussion

We present a case of TGCT of the hip in a 12-year-old male patient, who presented with acute, atraumatic hip pain with limited mobility, elevated ESR and CRP, and an effusion noted on radiographs and ultrasonography.

TGCT is an uncommon neoplastic disease that typically presents with chronic, insidious joint pain. In a study of 166 cases of TGCT, the average duration of symptoms before discovery was approximately 10 months, with no cases of acute symptoms.^4^ TGCT usually appears in the third to sixth decades of life.^3^ However, there have been few reports of TGCT in children in the literature, most of which involve the knee. A case series reviewed six children of ages 7 to 15 years with TGCT of various joints, most commonly the knee. In five of the six cases, the diagnosis was not suspected clinically or radiographically, leading to delay in treatment—in one case, as long as 2 years.^7^ Another case series of 17 pediatric patients, followed up over 16 years, demonstrated that most of the cases were misdiagnosed as various other rheumatologic or orthopaedic conditions, which the authors attribute to failure to consider TGCT as a possible diagnosis in these pediatric patients and an equivocal presentation of symptoms and examination findings.^14^ Both series reported that most patients had chronic joint pain or effusion, usually greater than 1 year.^7,14^

However, there have been few reports of TGCT in patients reporting of acute-onset hip pain, as in our patient. In the case series mentioned earlier, only 5 of the 17 patients had involvement of the hip, and of those five patients, only one had acute pain.^14^ In addition, two separate case reports described pediatric patients who both presented with acute, atraumatic hip pain, most concerning for septic hip. Given the acuity of the situation in both cases, open irrigation and mass excision were done, which both confirmed the diagnosis and treated the underlying pathology.^8,9^

The acute nature of our patient's hip pain may have been precipitated by necrosis of the tumor. There are only a few documented cases of necrotic TGCT in the literature, in which the patients all reported of acute-onset joint pain.^6,15-17^ In these cases, the hypothesized mechanism is torsion of the tumor pedicle and ultimately strangulation, compromising blood flow to the mass and leading to eventual necrosis. Given the similar acuity and pathology findings in this case, it is possible that this patient experienced a similar phenomenon.

When a child presents with acute, atraumatic joint pain with inability to bear weight and pain with joint motion, ruling out septic arthritis is paramount. Delay in treatment of a septic joint, especially a larger joint such as the hip, can lead to rapid deterioration or necrosis of the articular surface.^18,19^ When septic arthritis of the hip is suspected, the modified Kocher criteria are typically used.^20^ This patient presented with three of five criteria based on his inability to bear weight on the affected side and elevated ESR and CRP (>40 mm/hr and >2 mg/dL, respectively), which translates to an 83% probability of septic arthritis of the hip according to Caird et al.

Given the concern for septic arthritis in this patient, an arthrocentesis was done. Types of joint effusion can be categorized into four types—noninflammatory, inflammatory, septic, and hemorrhagic—each with their own criteria based on the synovial fluid analysis (Table 1).^21,22^ As in this case, arthrocentesis from previously reported cases of TGCT typically yielded bloody fluid with a high red blood cell count, variable WBC count, and a negative Gram stain, most consistent with hemorrhagic synovial fluid.^6,9^ With borderline results from serology and synovial fluid and an examination that was not entirely consistent with septic arthritis, other causes for joint pain must be suspected as well.

**Table 1:** Classification of Synovial Fluid

	Normal	Noninflammatory	Inflammatory	Septic	Hemorrhagic
Clarity	Transparent	Transparent	Translucent/opaque	Opaque	Bloody
Color/gross appearance	Colorless	Yellow	Yellow	Yellow/variable	Red
White blood cells (cells/mm^3^)	<200	<2,000	2,000-75,000	>50,000	Variable
Gram stain and culture	Negative	Negative	Negative	Usually positive	Negative

Imaging, such as MRI, helps delineate the differential diagnoses for atypical causes of joint pain, and in this case, raised suspicion for TGCT. However, at many institutions, MRI is not obtained before open irrigation and débridement for suspected septic arthritis, usually related to MRI availability. Delaying MRI until after an irrigation and débridement would have likely delayed or even missed this diagnosis, as a capsulotomy and irrigation and débridement—which is often done rather than a true débridement—would likely not visualize the intra-articular mass. Given the equivocal nature of this patient's symptoms and arthrocentesis, MRI before operating was vital to achieve an accurate, early diagnosis of TGCT and proper treatment. To not miss this diagnosis in pediatric patients, MRI should be obtained before surgical irrigation and débridement if there is an equivocal history and examination and hemorrhagic, inflammatory range, or nondiagnostic synovial fluid joint aspirations.

Because this patient's MRI was suggestive of TGCT, he underwent CT-guided biopsy to attempt to provide a definitive diagnosis before open resection, recognizing the potential implications should a sarcoma diagnosis render. However, the CT-guided biopsy was insufficient for diagnosis, and the patient ultimately underwent open biopsy with wide excision and partial synovectomy. CT-guided biopsies have a high reported diagnostic accuracy of 71% to 93%, compared with 98% for open biopsies.^23,24,25 26^ However, CT-guided biopsy is the preferred initial step to acquire tissue for diagnosis given its less invasive nature, shorter sedation and recovery times, and lower rates of complications compared with open biopsy. Nevertheless, 5% to 31% of image-guided biopsy can be nondiagnostic, and this may require repeat needle biopsy or open biopsy as in this case.^23,24^ In light of the index of suspicion, the oncologic team was prepared for en bloc excision of the mass, with intraoperative frozen section availability, to mitigate additional oncologic concerns.

## Conclusion

This case report identifies one of the few reported pediatric cases of TGCT presenting as an acute, irritable hip mimicking septic arthritis. Septic arthritis is of paramount concern in these presentations, and an infectious workup involving inflammatory markers, WBC count, and possibly arthrocentesis should be pursued. However, in patients with an equivocal examination and laboratory test results, TGCT—although uncommon—should be considered as part of the differential, and MRI of the affected joint should be obtained before surgical intervention to avoid delays in diagnosis and treatment of this pathology.
